# The effects and mechanisms of natural products on *Helicobacter pylori* eradication

**DOI:** 10.3389/fcimb.2024.1360852

**Published:** 2024-02-28

**Authors:** Ruiyi Deng, Xu Chen, Shiqing Zhao, Qingying Zhang, Yanyan Shi

**Affiliations:** ^1^ Research Center of Clinical Epidemiology, Peking University Third Hospital, Beijing, China; ^2^ Peking University First School of Clinical Medicine, Peking University First Hospital, Beijing, China; ^3^ Department of Natural Medicines, School of Pharmaceutical Sciences, Peking University Health Science Center, Beijing, China

**Keywords:** *Helicobacter pylori*, eradication, antibiotic resistance, ethnomedicine, natural products, complementary therapy

## Abstract

*Helicobacter pylori* (*H. pylori*) eradication is pivotal for alleviating gastric mucosal inflammation and preventing the progression of gastric diseases. While antibiotic-based therapies have achieved significant success in *H. pylori* eradication, challenges such as antibiotic resistance, drug toxicity, side effects, nonadherence, inapplicability, and disruption of gastrointestinal microflora have emerged. Updated therapies are urgently needed to suppress *H. pylori*. Nature has provided multitudinous therapeutic agents since ancient times. Natural products can be a potential therapy endowed with *H. pylori* eradication efficacy. We summarize the basic information, possible mechanisms, and the latest research progress of some representative natural products in *H. pylori* eradication, highlighting their safety, accessibility, efficiency, and ability to overcome limitations associated with antibiotic application. This review highlights the potential therapeutic advantages of incorporating ethnomedicine into anti-*H. pylori* regimens. The findings of this review may provide insights into the development of novel natural products and expand the therapeutic options available for *H. pylori* eradication.

## 1 Introduction

*Helicobacter pylori* (*H. pylori*) infection is a major cause of gastric mucosa injury. Approximately 1% of infected individuals will progress to gastric cancer (de Vries et al., 2008). Therefore, the WHO classified *H. pylori* as a class I carcinogen in 1994 (Cancer, 1994). According to statistics, approximately half of the world’s population is affected by *H. pylori* infection, which accounts for 15% of the global cancer burden (Shah et al., 2021). Therefore, this pathogen exerts a profound influence on society and the economy, and it is crucial to find an effective method for *H. pylori* eradication to avoid potential accompanying diseases. Some risk factors have been reported to be associated with *H. pylori* infection, such as smoking, unboiled water and uncooked food intaking, poor socioeconomic conditions, and early childhood exposure to the bacterium (Leja et al., 2019; Yuan et al., 2022). Antibiotic combination therapy is needed to eradicate *H. pylori*, for example, the standard PPI-clarithromycin-containing triple therapy and bismuth quadruple therapies (Malfertheiner et al., 2017). However, in the context of a global increase in antibiotic consumption, antibiotic resistance of *H. pylori* has reached a crisis point, which poses a severe threat to the current regimens (Fallone et al., 2016). Consequently, clarithromycin-resistant strains of *H. pylori* were recognized by the WHO in 2017 as one of 12 priority pathogens in urgent need of novel antibiotics or alternatives (Tacconelli et al., 2018). As a result, new therapeutic therapies are imperative.

**Figure 1:**
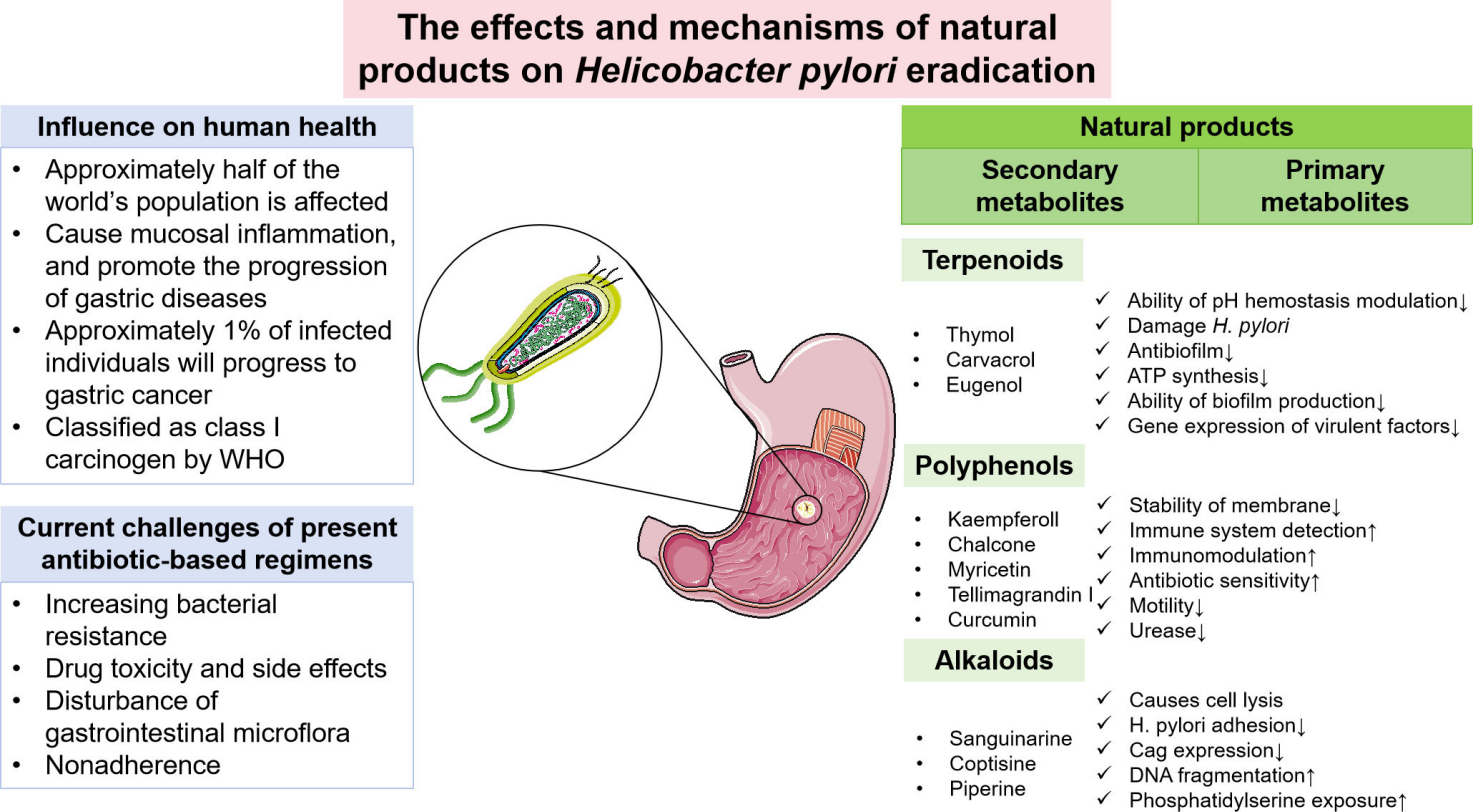
Current challenges of *H. pylori* eradication and the potential role of natural products in eradicating *H. pylori*.

Complementary and alternative treatments with features of lower toxicity and low cost are attractive, and natural compounds have long been viewed as vital candidates for anti-*H. pylori* regimens. Researchers in gastroenterology or bacteriology domains have shown remarkable interest in the antimicrobial activities of natural products, especially for the possible lessened likelihood of developing genetic resistance. With a view to identifying more medicinal natural products and their synthetic variations, it is ideal to take a deep look into ethnomedicine, for example, traditional Chinese medicine (TCM). Numerous *in vitro* and *in vivo* studies have been conducted to investigate the therapeutic effect of ethnomedicine and their corresponding natural products against *H. pylori*. For incidence, the *in vitro* efficacy of artemisone and artemisinin derivatives against *H. pylori* was demonstrated in the work by Sisto et al (Sisto et al., 2016), while it also indicated a synergistic effect combined with standard drugs. In addition, Krzyzek et al. identified myricetin as a natural substance that hampers the morphological transition of *H. pylori* from spiral to coccoid forms and therefore increases susceptibility to antibiotics (Krzyżek et al., 2021). Confronting the antibiotic resistance affecting *H. pylori* eradication, such studies elucidate applicable natural products as alternative therapies. Great progress has been made in this field, but few natural product-based regimens have been recommended by clinical guidelines, and the mechanisms behind ethnomedicine are still not clearly explained. Consequently, the keywords “*H. pylori*”, “natural products” and “ethnomedicine” were comprehensively searched in PubMed, Wed of Science databases, China National Knowledge Infrastructure (CNKI), and Scopus databases. The publications were screened and those related to the theme of this review were included. Based on these relevant studies, we summarized the general situation and possible therapeutic mechanisms of natural products potentially with anti-*H. pylori* properties to serve as a reference for the development of novel drugs and expand therapeutic options against the bacterium (**Figure 1**).

## 2 Physiological properties and pathogenic mechanisms of *H. pylori*

*H. pylori* is a gram-negative, spiral, and microaerophilic bacterium that was discovered by Australian scientists Warren and Marshall in 1982 (Marshall and Warren, 1984). While the majority of bacteria are incapable of colonizing in such an acidic environment, *H. pylori* has evolved unique features that allow it to thrive in the exclusive ecological niche of the human stomach (Ansari and Yamaoka, 2019). These features include its mobility, helical shape, and ability to produce large amounts of urease, which neutralizes gastric hydrochloric acid by hydrolyzing urea into bicarbonate and ammonia (Solnick and Schauer, 2001). Furthermore, *H. pylori* possesses a chemotaxis system that enables it to detect pH gradients as well as sheathed polar flagella, which aid in reaching less acidic gastric mucosal surfaces (Roszczenko-Jasińska et al., 2020). Once there, adhesins such as blood-antigen binding protein A (BabA) facilitate the attachment of the bacteria to gastric epithelial cells (Sousa et al., 2022). In addition to factors involved in establishing infections, many clinical isolates of *H. pylori* produce virulence factors such as VacA, CagA, and cagPAI within the host cell cytoplasm. These factors play significant roles in immune evasion and disease induction (Sukri et al., 2020). Infected gastric tissues also exhibit elevated levels of reactive oxygen species (ROS), leading to gastric inflammation with the production of various mediators (Kang and Kim, 2017).

## 3 General status, treatment guidelines, and current challenges of *H. pylori* eradication

*H. pylori* eradication is reported to help alleviate mucosal inflammation, restore normal mechanisms governing acid secretion, and stop the progression of gastric diseases (Sugano et al., 2015). Accordingly, the present consensus among all major gastroenterological societies is that *H. pylori* infection should be eradicated in individuals who tested positive unless there are compelling reasons (Sugano et al., 2015; Matsumoto et al., 2019; Shah et al., 2021). Since the microorganism was discovered, a wide variety of therapeutic strategies have been proposed to tackle *H. pylori*, such as clarithromycin triple therapy, bismuth or nonbismuth-based quadruple therapy, and rifabutin-based triple therapy (Cardos et al., 2021).

The option of eradication regimes should be determined according to the geographical area, in the context of regional differences in antibiotic resistance patterns, as well as the different common host genotypes of drug-metabolizing enzymes of the local population. In regions with low clarithromycin resistance (<15%), the Maastricht consensus report still endorses standard triple therapy as the primary treatment option (Malfertheiner et al., 2017), which consists of a proton pump inhibitor (PPI), amoxicillin, and clarithromycin or metronidazole. However, the regime is inapplicable in high clarithromycin resistance regions without preceding antimicrobial susceptibility testing. Metronidazole is an ideal substitute for clarithromycin in regions with low to intermediate metronidazole resistance rates (Malfertheiner et al., 2017). When high resistance to both antibiotics is observed, bismuth quadruple or nonbismuth quadruple, concomitant (PPI, amoxicillin, clarithromycin, and nitroimidazole) therapies are favorable options instead (Sugano et al., 2015; Malfertheiner et al., 2017).

Despite the significant progress made by antibiotic-based therapies, the present regimens are faced with a series of challenges, for example, high resistance to antibiotics, nonadherence, drug toxicity and side effects, and disturbance of gastrointestinal microflora (Matsumoto et al., 2019).

Increasing bacterial resistance to commonly used antimicrobial agents is one of the most common reasons for eradication failure, which has reached alarming levels worldwide and severely threatens the efficacy of treatment. Based on a systematic review and meta-analysis involving 65 countries, the incidence of primary and secondary resistance to clarithromycin, metronidazole, and levofloxacin is greater than 15% in most WHO regions (Savoldi et al., 2018). This threshold is commonly used to select alternative treatment regimens (Savoldi et al., 2018). Likewise, a recent prospective study covering 18 European countries also showed alarming *H. pylori* resistance to commonly used antibiotics and revealed a positive correlation between macrolide and quinolone consumption and corresponding *H. pylori* resistance (Megraud et al., 2021). Accordingly, the last few decades have witnessed the unacceptable fact that *H. pylori* eradication rates of standard triple therapies experienced a sharp decrease from 80-90% in the 1990s to below 70%, and other regimens also encounter similar hurdles (O'Morain et al., 2018). To maximize the eradication rate and ensure the prudent use of antibiotics, individual antibiotic susceptibility testing seems to be a feasible solution, which is especially recommended in cases needing salvage therapies (O'Morain et al., 2018). Moreover, it is beneficial to collect regional antibiotic consumption data and clinical outcomes to provide local resistance reports and update guidelines for infection (Matsumoto et al., 2019).

In addition to the emergence of antibiotic-resistant strains, the possible adverse effects and nonadherence of some patients also impose restrictions on the utilization of conventional antibiotic-based eradication therapies. Antibiotics, especially at high dosages, may induce many side effects. For example, antibiotics are known to affect the balance of gut microbiota through both direct and indirect mechanisms. While eradicating targeted pathogens, antibiotics also discriminately kill or inhibit subsets of commensal microbes or disrupt the homeostasis of the symbiosis and codependency relationships among different subsets of gut microbiota (Zhang et al., 2019). In addition, some individuals may experience adverse reactions, including nausea, allergic reactions to antibiotics, and severe complications (liver and/or kidney dysfunction) (Takeuchi et al., 2014). The level of patient compliance is another crucial contributing factor for treating the infection, which can be influenced by barriers such as physical intolerance of medication, lack of understanding of the prescription, and high pill burden (Shah et al., 2021). The threshold of adherence for successful eradication varies depending on individual factors, but it was demonstrated that patients need to hold to at least >60% to >90% of the prescribed course to guarantee the eradication rate (Shah et al., 2021). Furthermore, host genetic polymorphisms of drug-metabolizing enzymes also account for the eradication failure of *H. pylori* infection. The metabolism-enhancing phenotypes of CYP2C19 are responsible for the rapid metabolism of some PPIs and thus influence the reduction in intragastric acidity. The optimal intragastric pH for *H. pylori* replication is between 6 and 8; thus, alkalizing the gastric environment can increase susceptibility to antibiotics in populations with metabolism-enhancing phenotypes of CYP2C19 (Safavi et al., 2015; Shah et al., 2021).

Due to the current persistence and rise of antibiotic resistance and other restrictions on current therapies, traditional antibiotic-based therapies have been severely weakened, and the development of novel and more effective antimicrobial compounds and novel strategies is drawing increasing attention worldwide (Matsumoto et al., 2019). However, owing to the complex biology of the pathogen, the high cost of research and the lack of financial support, the discovery and development of new therapeutics is not without challenges. To reduce the cost of developing new candidates for *H. pylori* eradication, it is beneficial to attach importance to the exploitation of natural product libraries and conduct thorough ethnobotanical and ethnomedical analyses, which may offer valuable inspiration for new treatments.

## 4 Natural products effects in *H. pylori* eradication and their healing mechanisms

### 4.1 The role of natural products and ethnomedicine

In light of the challenges faced by currently used *H. pylori* antibiotic-based eradication therapies, research on alternative treatment approaches is gaining popularity. With vast resources, nature has provided multitudinous therapeutic agents since ancient times and is seen as a promising source in the search for new compounds endowed with anti-*H. pylori* potential.

Ethnomedicine is the study of naturally obtained drugs based on traditional knowledge and practices of various ethnic groups (Lai et al., 2022), usually supported by ancient medical classics or passed down orally over generations. Generally, acquired from empirical observations and beliefs of indigenous peoples, the theoretical systems of ethnomedicine differ from scientific medicine, and some are still currently inexplicable by science. Nevertheless, ethnomedicine has been indispensable in health maintenance in households for centuries and is still widely used for primary health needs in extensive regions (Buenz et al., 2018).

Natural products are a series of bioactive chemical compounds produced by living organisms from nature, usually serving as the primary and secondary metabolites in biochemical pathways (Katz and Baltz, 2016). Typical examples of natural products include alkaloids, flavonoids, saponins, terpenes, etc. Although not universally recognized, natural products are widely acknowledged as the active components of numerous ethnomedicines, and the pertinent cutting-edge research findings have input vigor into the domain of drug discovery and development.

Several natural products and ethnomedicine have demonstrated antimicrobial activity through diverse scientific research as well as multiple generations of medical practice. Even before the discovery of *H. pylori*, a wide range of plants and substances have been employed to address gastric symptoms that are currently considered to be associated with the infection of this pathogen (Takagi and Harada, 1969; Escobedo-Hinojosa et al., 2012). Today, with a more in-depth knowledge of *H. pylori* after decades of exploration, ethnomedicine is still being used as a guide for the search for effective natural products and treatment approaches to cope with the infection (Vale and Oleastro, 2014), and some patent medicines have already been recommended as supplements in clinical guidelines. For example, traditional Chinese medicine and Kampo are recommended as treatment options by countries with prevalent use of traditional medicine (Helicobacter pylori Study Group, 2022; Miwa et al., 2022). The bioactivity of some natural products in inhibition of *H. pylori* has been summarized in **Table 1**.

**Table 1:** Summarization of the bioactivities of natural products in *H. pylori* inhibition.

Compounds name	MIC (μg/mL)	MBC (μg/mL)	*H. pylori* strains	Reference
Limonene	75	/	ATCC 43504	(Rozza et al., 2011)
	199.2	/	ATCC 43504	(Villa-Ruano et al., 2018)
β-pinene	500	/	HATCC 43504	(Rozza et al., 2011)
	296.9	/	ATCC 43504	(Villa-Ruano et al., 2018)
α-pinene	/	100	P1	(Bergonzelli et al., 2003)
β-Myrcene	500	/	ATCC 43504	(Bonamin et al., 2014)
Thymol	64-128	/	Eight strains of *H. pylori* (one reference and seven clinical isolates)	(Sisto et al., 2021)
Carvacrol	16–64	32–64	Nine strains of *H. pylori* (one reference strain and eight clinical isolates)	(Sisto et al., 2020)
Eugenol	23.0-51.0	/	*H. pylori* isolates and standard strain NCTC 11637	(Elbestawy et al., 2023)
Glycyrrhetinic acid	50	/	29 different *H. pylori* strains	(Krausse et al., 2004)
Kaempferol	>600	/	ATCC 43504 and 26695	(Escandón et al., 2016)
Chalcone (Xinjiachalcone A)	12.5-50	/	Seventeen *H. pylori* strains including clinical ones	(Bodet et al., 2014)
Myricetin	160	320	ATCC 700824, 51932	(Krzyżek et al., 2021)
Taxifolin	625	/	ATCC 43629	(Stenger Moura et al., 2021)
Tellimagrandin I	12.5	/	NCTC 11638, ATCC 43504, A-13, A-19	(Funatogawa et al., 2004)
Tellimagrandin II	6.25-12.5	/	NCTC 11638, ATCC 43504, A-13, A-19	(Funatogawa et al., 2004)
Curcumin	30	40	ATCC 700392	(Darmani et al., 2020)
Berberine	25	/	NCTC 26695 and SS1	(Wu et al., 2023)
Coptisine	16-32	32-64	ATCC 43504,700392 and PMSS1	(Tang et al., 2023)
Palmatine	50	/	NCTC 26695 and SS1	(Wu et al., 2023)
	32-64	64-128	ATCC 43504,700392 and PMSS1	(Tang et al., 2023)
Sanguinarine	6.25–50	/	A total of 14 clinical isolates	(Mahady et al., 2003)
Piperine	125	/	60190, NTCC 11637 and Tx30a	(Tharmalingam et al., 2014)

### 4.2 The mechanisms of natural products in *H. pylori* eradication

Natural products are usually divided into two categories: primary metabolites and secondary metabolites. Including carbohydrates, lipids, proteins, and nucleic acids, primary metabolites are indispensable and directly involved in the life cycle of organisms (Chaturvedi and Gupta, 2021). Few of them, however, demonstrate pharmacologic actions against microorganisms. Several polysaccharides have exhibited antiadhesive properties against *H. pylori*, likely by inhibiting the crucial docking process of adhesins of *H. pylori* and gastric epithelium (Gottesmann et al., 2020).

**Figure 2:**
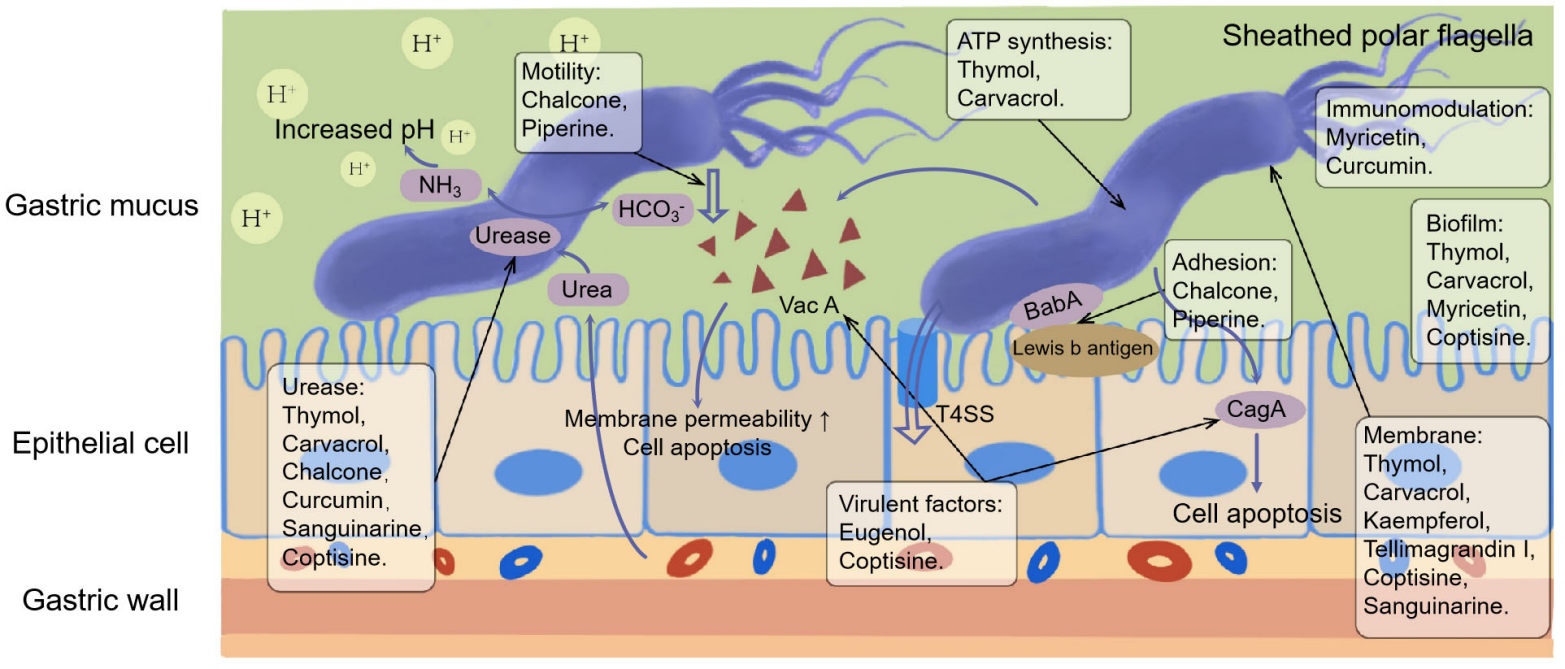
Mechanisms of secondary metabolites for *H. pylori* eradication.

In contrast, secondary metabolites are compounds that are not needed for life, usually not only possessing a wide range of biological functions but also having special mechanisms of action (Li et al., 2021). The scientific community has invested significant expectations in the utilization of secondary metabolites, and a large number of studies have been performed to reveal the pharmacological value of these compounds. We summarized the basic information and possible mechanisms of some representative secondary metabolites for *H. pylori* eradication (**Table 2** and **Figure 2**). The section below describes the latest research progress of secondary metabolites for *H. pylori* eradication.

**Table 2:** Summarization of the basic information and possible mechanisms of some representative secondary metabolite for *H. pylori* eradication.

Compounds name	Reported from (plant name)	Mechanisms	Reference
Terpenoids			
**Thymol** 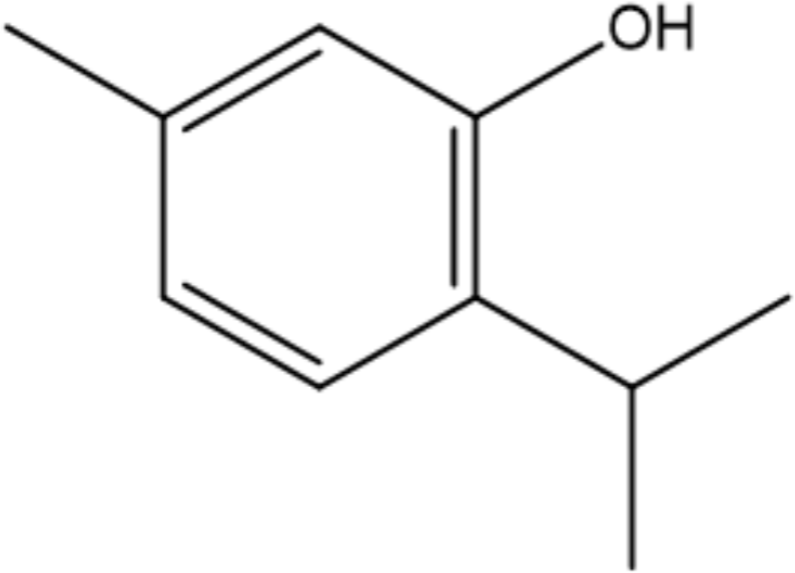	*Thymus kotschyanus*	Ability of pH hemostasis modulation↓; damage *H. pylori*; membrane integrity; ATP synthesis↓; ability of biofilm production↓.	(Grande et al., 2021; Sisto et al., 2021)
**carvacrol** 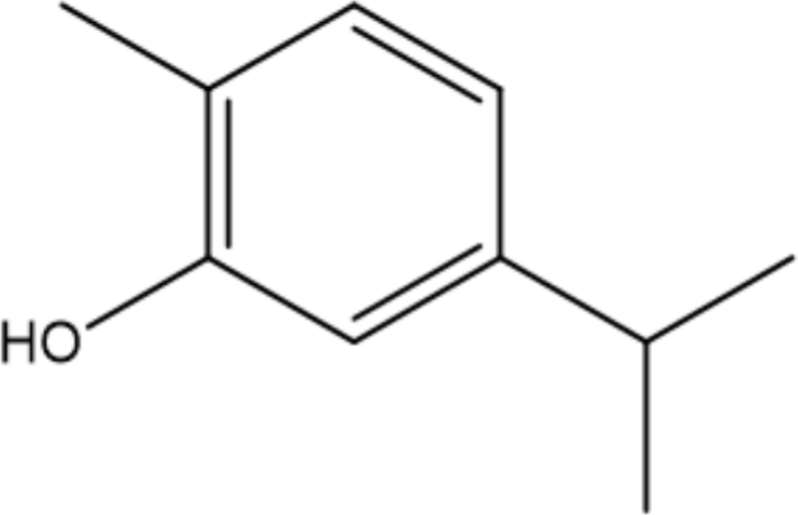	*Origanum vulgare*	Ability of pH hemostasis modulation↓; damage *H. pylori*; membrane integrity; ATP synthesis↓; ability of biofilm production↓.	(Oliveira et al., 2012; Sisto et al., 2020; Grande et al., 2021)
**Eugenol** 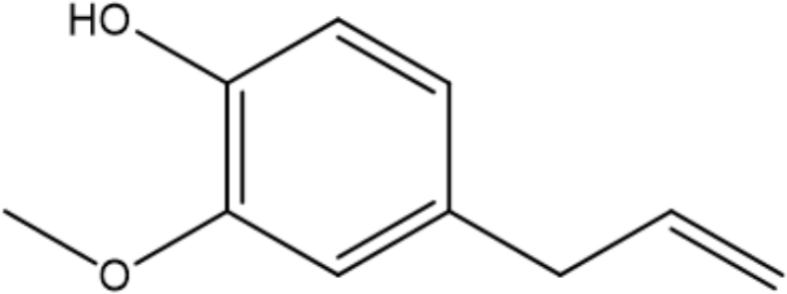	*Eugenia caryophillis*	Antibiofilm↓; gene expression of virulent factors↓;	(Wei and Shibamoto, 2010; Hu et al., 2018; Elbestawy et al., 2023)
Polyphenols			
**kaempferol** 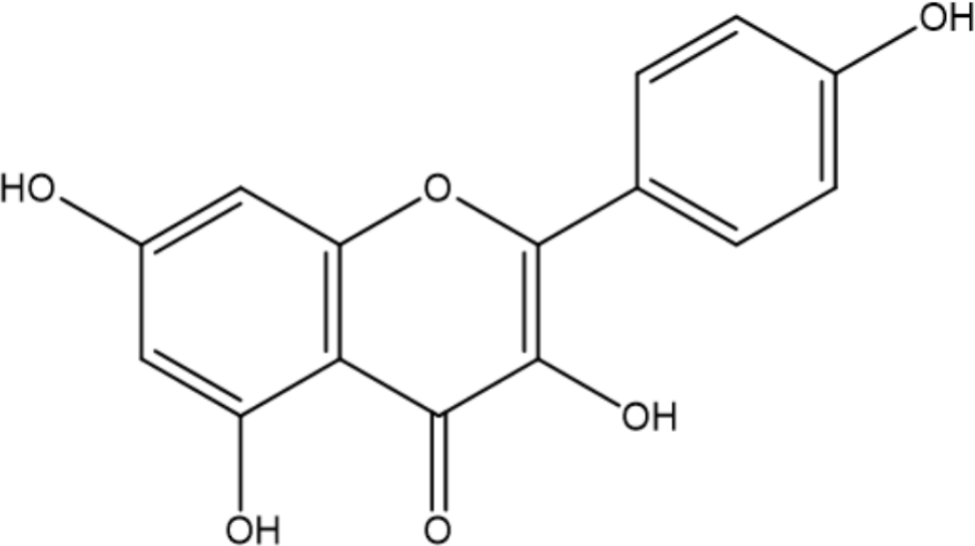	*kaempferol galanga*	Stability of membrane↓.	(Yang et al., 2022)
**chalcone** 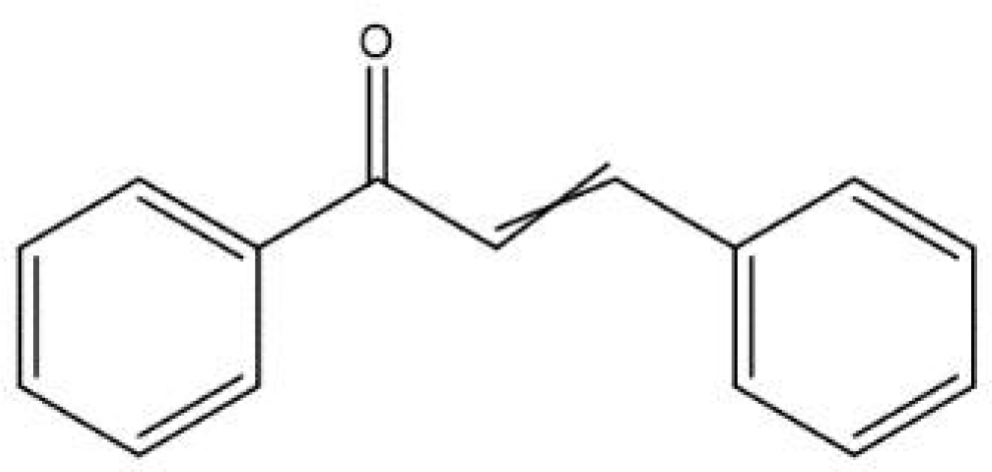	species of the *Leguminosae, Asteraceae and Moraceae* families	Interactions between *H. pylori* and gastric epithelium↓; motility↓; urease↓;	(Michalkova et al., 2023)
**myricetin** 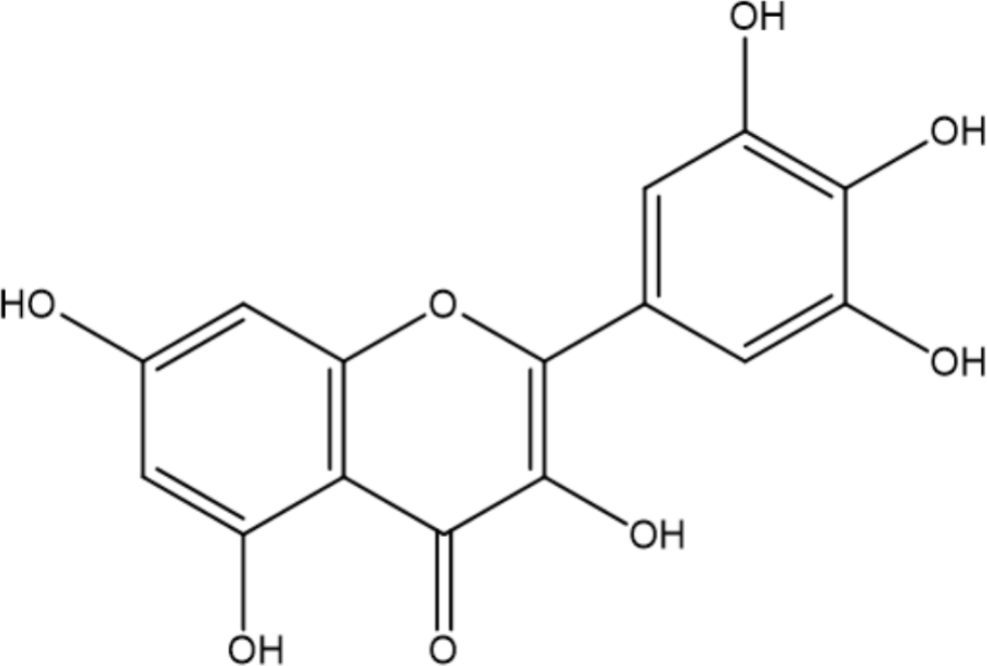	*Myrica*	Immune system detection↑; antibiotic sensitivity↑; biofilm↓.	(Krzyżek et al., 2021)
**Tellimagrandin I** 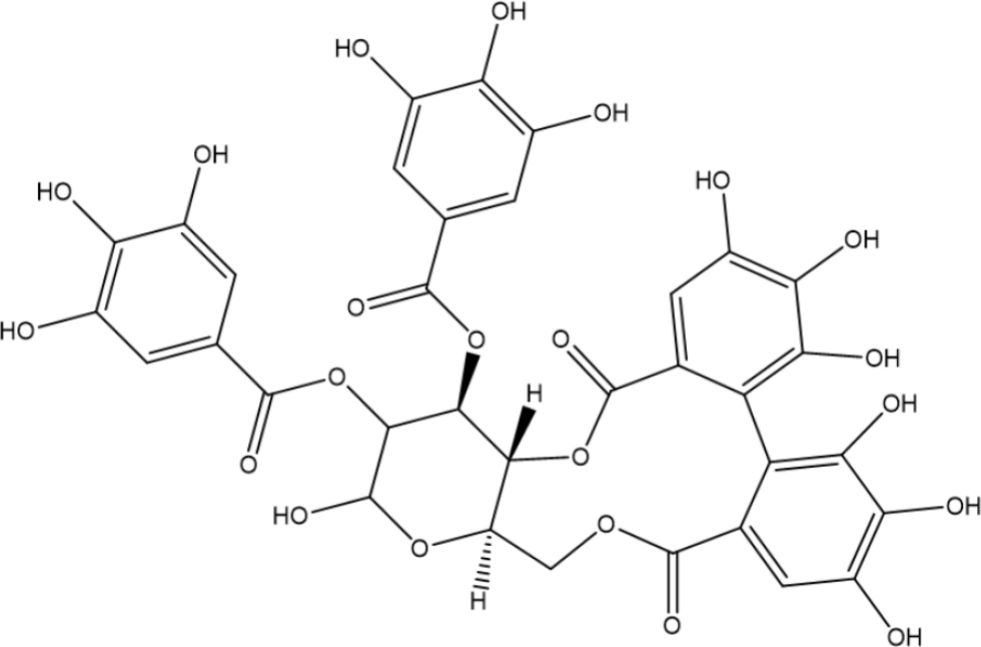	*Cornus canadensis*	damage *H. pylori*; membrane integrity;	(Funatogawa et al., 2004)
**Curcumin** 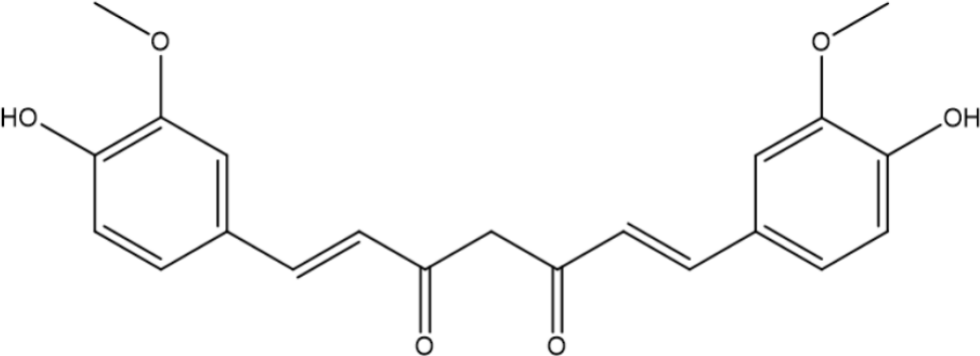	*Turmeric*	Urease↓; immunomodulation↑.	(De et al., 2009; Khonche et al., 2016; Judaki et al., 2017; Shetty et al., 2021)
Alkaloids			
**sanguinarine** 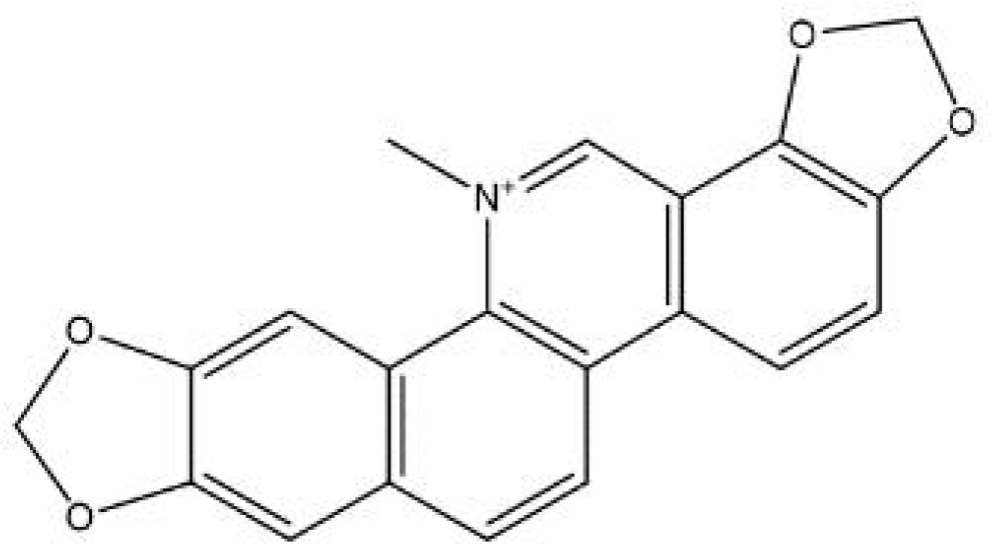	*Zanthoxylum nitidum*	Urease↓; cell lysis.	(Thawabteh et al., 2019; Lu et al., 2022)
**coptisine** 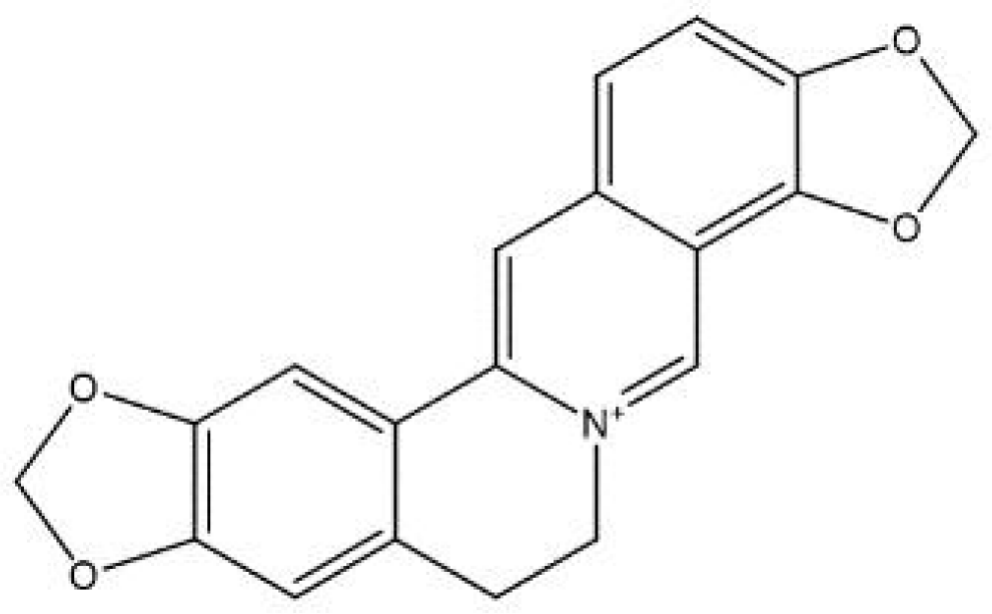	*Rhizoma Coptidis*	Urease↓; Cag expression↓; DNA fragmentation↑; phosphatidylserine exposure↑; membrane stability↓.	(He et al., 2022b; Tang et al., 2023)
**Piperine** 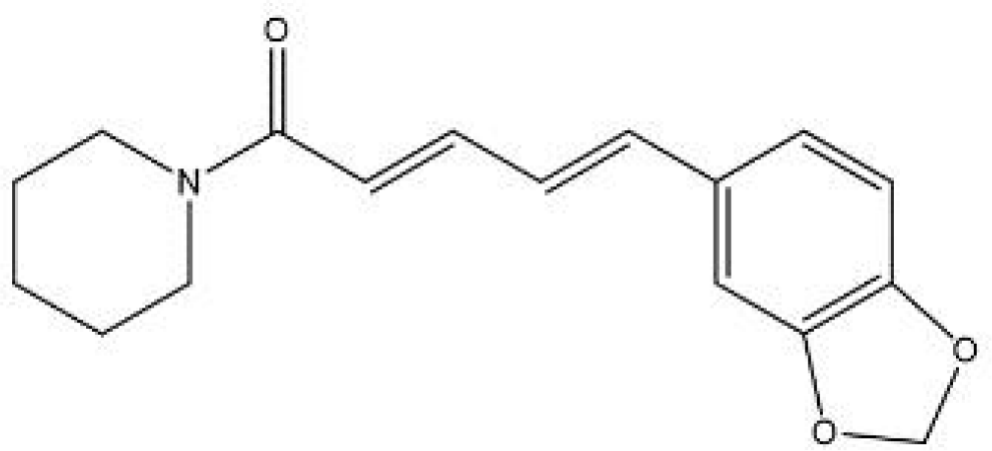	*black pepper*	Motility↓; *H. pylori* adhesion↓.	(Tharmalingam et al., 2014)

#### 4.2.1 Terpenoids

Terpenes are a large class of plant secondary metabolites formed with five-carbon isoprene (C_5_H_8_), and classifications of terpenoids are based on the number of isoprene units in their structure (Khan et al., 2018). As widely diffused chemicals in plants, many types of terpenoids have been shown to have anti-*H. pylori* properties, and here, we will update the reports of the antimicrobial effects of some terpenoids.

Monoterpenoids are a series of natural products that possess two isoprenes in their molecules, which are the prevailing components of the essential oils of pine, lemon, thyme, tea tree, etc., and many of them have demonstrated gastroprotective activity *in vivo* as well as antimicrobial properties *in vitro* (Rozza et al., 2011). Typical examples with anti-*H. pylori* properties include limonene and β-pinene, which are predominant components in *Citrus lemon* (Rutaceae) essential oil and have been demonstrated to exert antibacterial effects *in vitro*. Limonene was shown to elevate mucus secretion by ensuring adequate PGE_2_ levels, thus limiting the colonization of *H. pylori* and isolating gastric mucosa from ulcerative factors (Moraes et al., 2009). Additionally, monoterpenes demonstrate anti-inflammatory capacity by regulating oxidative stress and the inflammatory response of gastric mucus. Consequently, the expression of MPO, NF-κB, and proinflammatory cytokines such as TNF-a, IL-6, and IL-1β is decreased, while that of IL-10 is increased (de Souza et al., 2019). β-Pinene can also promote mucus production, relieve oxidative stress and inflammation and inhibit *Nf-κb* expression to exert its gastroprotective properties (de Souza et al., 2019). Similarly, α-pinene manifests gastroprotective properties with modulation of oxidative stress and PGE_2_ and histamine levels *in vivo* (Al-Sayed et al., 2021). β-Myrcene also possesses the capacity to block the growth of *H. pylori*, and the corresponding MIC of the monoterpene is 500 μg/mL (Wei and Shibamoto, 2010). For gastroprotective effects, β-myrcene prevents gastric damage by the marked upregulation of antioxidant enzyme activity, with decreased activity of superoxide dismutase (SOD) and increased levels of glutathione peroxidase (GPx), glutathione reductase (GR), and total glutathione (Bonamin et al., 2014). Moreover, terpenoid phenols, thymol and carvacrol, are reported antimicrobial agents with MIC ranges of 64-128 µg/mL and 16-64 µg/mL (Grande et al., 2021). A specific concentration of carvacrol or thymol is observed to cause an increase in membrane fluidity, leakage of essential ions, and limited ATP synthesis in pathogens, which is partly due to the hydrophobic character and may also be attributed to the presence of a hydroxyl group and a system of delocalized electrons in their structures, causing a decrease in the membrane pH gradient and thus interfering with ATP synthesis (Ultee et al., 2002). Additionally, the inhibition of carbonic anhydrase (CA), a series of enzymes that catalyze the equilibrium between CO_2_ hydration, may also play a crucial role in the antibacterial mechanism of the two terpenoids. *H. pylori* CAs are pivotal in the modulation of pH hemostasis, the integrity of the bacterial membrane, and the ability to produce biofilms, which are promising druggable targets in eliminating pathogenic microorganisms (Grande et al., 2021). Last, Eugenol is thought to be one of the most active natural products against *H. pylori*, with MICs ranging from 23.0 to 51.0 µg/mL (Elbestawy et al., 2023). Previous studies indicate that eugenol possesses antibiofilm activities against *H. pylori* and can downregulate the expression of virulence factors, while eugenol can also relieve *H. pylori*-related gastritis by its anti-inflammatory activities (Hu et al., 2018).

Sesquiterpenoids are another group of predominant secondary metabolites in various plant essential oils. For instance, over 70% of the components present in cedarwood essential oil are sesquiterpenoids, including α-, β-cedrene, thujopsene, cedrol, and cuparene, and the compound group displayed excellent efficacy in inhibiting urease activity and *H. pylori* growth (Korona-Glowniak et al., 2020), which may indicate that the anti-*H. pylori* activity of sesquiterpenoids. Additionally, with a similar structure to the major components in cedarwood essential oil, patchouli alcohol, a tricyclic sesquiterpenoid, exhibited antimicrobial properties toward *Streptococcus mutans* via the inhibition of DNA polymerase in a previous study (Takao et al., 2012), which may also offer a clue for the anti-*H. pylori* activity of sesquiterpenoids.

Similarly, triterpenoids have also gained attention for their various biological functions, including anti-inflammatory, antitumor, antiviral and antimicrobial activities (Dzubak et al., 2006). Triterpene glycosides (saponin) are good examples of anti-*H. pylori* activity of triterpenoids. For instance, glycyrrhizic acid is a representative triterpenoid saponin enriched in Glycyrrhiza glabra, whose major metabolite, glycyrrhetinic acid, was reported to exhibit rapid anti-*H. pylori* property *in vitro* (Wittschier et al., 2009). Glycyrrhetinic acid is reportedly in possession of cytotoxic effects and can impair *H. pylori* growth. Nevertheless, some studies have indicated that long-term intake of saponin may lead to an increased risk of gastric lesions (Périco et al., 2015). Therefore, the use of saponin-rich plants as an antiulcer ethnomedicine should be under careful consideration.

Tetraterpenoids are terpenoids that consist of a C40 structure, and carotenoids are a well-known subclass of tetraterpenoids that possess potent antioxidant properties. Previous research has explored the potential of some carotenoids in the treatment of *H. pylori*-related gastric diseases, including β-carotene (Bae et al., 2021), astaxanthin (Davinelli et al., 2019; Kim and Kim, 2021; Lee et al., 2022), and lycopene (Jang et al., 2012; Kim et al., 2018a). Carotenoids can be divided into two groups by the presence of oxygen, namely, xanthophylls and carotenes, and here, we will present β-carotene and astaxanthin as representatives. Abundant in orange-colored fruits and vegetables, β-carotene is a well-known carotenoid with marked antioxidant capacity, which may be structurally ascribed to the numerous conjugated double bonds (Kang and Kim, 2017). β-carotene intake can suppress NADPH oxidase and stimulate antioxidant enzyme activity, thus contributing to the suppression of *H. pylori*-induced ROS generation as well as the level of iNOS and COX-2 expression. As a result, β-carotene exerts its anti-inflammatory effects by suppressing ROS-mediated inflammatory signaling (including MAPKs and NF-κB), which functions in the prevention of inflammatory damage (Jang et al., 2009; Park et al., 2019; Bae et al., 2021). However, previous studies have mainly focused on the ability of β-carotene to modulate gastric carcinogenesis, and existing evidence has not shown explicit antimicrobial activity of β-carotene against *H. pylori*, which needs further exploration. In addition, astaxanthin is a xanthophyll carotenoid commonly found in crustaceans such as shrimp, crabs, and lobster (Davinelli et al., 2019), which is reported to have 10-fold stronger antioxidant activity than β-carotene. *In vivo*, a study using BALB/cA mice showed that an astaxanthin-rich algal meal could inhibit the colonization of *H. pylori* and suppress inflammation in gastric tissues of *H. pylori*-infected mice (Bennedsen et al., 2000; Naguib, 2000; Wang et al., 2000). Similarly, astaxanthin demonstrates its ability to prevent oxidative stress-mediated inflammation through ROS reduction (Kim et al., 2018b). Some research also illustrated that astaxanthin could induce a shift in the Th1/Th2 response pattern, thus enhancing the clearance of *H. pylori* (Davinelli et al., 2019). Additionally, a significant ability to inhibit H^+^, K^+^-ATPase was observed in astaxanthin esters, indicating a possible mechanism of *H. pylori* inhibition and a potent future of astaxanthin modification (Kamath et al., 2008).

#### 4.2.2 Polyphenols

Polyphenols are another ubiquitous spectrum of natural products abundant in green tea, propolis, cranberry, etc (Gao et al., 2021; Ngan et al., 2021; Widelski et al., 2022), and can be grouped into classes such as flavonoids and tannoids. Structurally, polyphenols possess several hydroxyl groups on aromatic rings, which contribute to their antioxidant properties to reduce ROS production (Bonacorsi et al., 2012). Several investigations into the mechanisms of anti-*H. pylori* capacities have shown that polyphenols may lead to a decrease in urease activity, inhibition of cytotoxic activity as well as its binding with the gastric mucosa, and the rupture of outer membrane (Burger et al., 2002; Lin et al., 2005; Yahiro et al., 2005; Nohynek et al., 2006). According to a previous study, polyphenols as an adjuvant in the treatment of *H. pylori* infection can significantly improve the eradication rate (Wang et al., 2023; Wu et al., 2023), while no evidence has been found to suggest an increased risk of side effects. Consequently, polyphenols are suggested as an alternative treatment approach for *H. pylori* infection.

Among the polyphenol family, flavonoids are one of the most important and vast groups of compounds with a basic skeleton comprising two benzene rings linked through a heterocyclic ring, involving over 9000 species of molecules (Gonzalez et al., 2021). Many flavonoids have been shown to possess promising antimicrobial capacity against *H. pylori*, including kaempferol, chalcone, myricetin, taxifolin, and other compounds with proven validity (Krzyżek et al., 2021; Stenger Moura et al., 2021; Yang et al., 2022; Michalkova et al., 2023). Flavonoids contribute to several antibacterial mechanisms, such as the inhibition of crucial enzymes for colonization, survival, and reproduction (Asha et al., 2013; Steinmann et al., 2013; Egas et al., 2018), and interference with the fluidity and stability of the cytoplasmic membrane (Tsuchiya and Iinuma, 2000; Yang et al., 2022). In addition, flavonoids can engage in the modulation of several intracellular pathways, such as MAPK and NF-κB, thus attenuating the level of pro-inflammatory cytokines induced by *H. pylori* infection and improving gastric inflammation status (Wang and Huang, 2013). In addition to their antimicrobial and anti-inflammatory effects, flavonoids are capable of acting synergistically with antibiotics commonly utilized in the treatment of *H. pylori* infection (Krzyżek et al., 2021).

Tannoids (or tannins) are naturally occurring plant polyphenolic substances that bind to proteins, amino acids, alkaloids, and precipitate them and are highly expected antimicrobial biomolecules (Kurhekar, 2016), and previous studies have demonstrated the *in vitro* anti-*H. pylori* activity of the compounds (Wang, 2014; Cardoso et al., 2018). According to Funatogawa et al.’s work, monomeric hydrolyzable tannoids inclusive of tellimagrandin I and II exhibit strong antibacterial activity by damaging the membrane of *H. pylori* (Funatogawa et al., 2004). In addition, tannoids can also serve as inflammatory mediators by reducing nitric oxide levels and exerting anti-inflammatory activity on gastric mucosa (Cardoso et al., 2018).

Curcumin, the principal ingredient isolated from *turmeric*, has been used in many Asian regions as an herbal remedy for various diseases (Kwiecien et al., 2019). *In vivo*, an experiment in a mouse model has proven a notably reduced number of *H. pylori* colonizing mucosa, which may be attributed to decreased activity of lipid peroxide, MPO and urease and increased level of immunomodulation (De et al., 2009). Recent evidence offered by randomized clinical trials also revealed that triple therapy with curcumin can improve symptoms of dyspepsia, attenuate oxidative stress, and prevent mucosa against inflammation, and some showed that adjunctive therapy with curcumin can improve the eradication rate of *H. pylori* (Khonche et al., 2016; Judaki et al., 2017). Additionally, the good interaction between curcumin and targeted virulence factors such as Ureα/β subunits is implicated in the prevention of *H. pylori* survival and colonization (Shetty et al., 2021). However, the oral bioactivity of curcumin as an alternative remedy for *H. pylori* elimination is weakened by its limited aqueous solubility and retention time, for which an effective delivery system is needed to ensure enough therapeutic window at the site of infection, and chitosan (CS) polymer may be a practical solution (Ejaz et al., 2022).

#### 4.2.3 Alkaloids

Alkaloids are a wide range of naturally occurring molecules that contain at least one nitrogen atom and several hydrogen-carbon groups in their structures, forming heterocyclic rings. To date, over 10,000 kinds of alkaloids have been identified among 300 plant families (Mahapatra et al., 2019). A variety of compounds in alkaloid families have shown multiple therapeutic effects, for example, their antitumor, antimicrobial, anti-inflammatory and antidiabetic properties (Wu et al., 2018). Numerous previous studies have noted diverse representatives of pharmaceutically used alkaloids, among which some are recognized as promising antimicrobial agents, such as berberine, coptisine, palmatine, and quinolone alkaloids (Hamasaki et al., 2000; Wen et al., 2022). Some studies have focused on the mechanisms of anti-*H. pylori* properties, which will be discussed in the following section.

The inhibition of urease activity is a common mechanism of alkaloids against *H. pylori*. According to the significant reduction in anti-urease activity after adding sulfhydryl-containing reagents or competitive Ni^2+^-binding restrainers, sanguinarine (a natural alkaloid enriched in Zanthoxylum nitidum) is supposed to suppress urease by targeting Ni^2+^ and thiol (Lu et al., 2022). Similarly, coptisine could also interact with both active center Ni^2+^ and sulfhydryl groups in amino acid residues to inhibit urease activity (He et al., 2022b). In addition to urease inhibition, coptisine also demonstrates various antibacterial mechanisms, including decreasing Cag expression and inducing DNA fragmentation (Tang et al., 2023). Moreover, many alkaloids, such as coptisine and squalamine, can cause disruption of the cell membrane of *H. pylori*, which is especially beneficial for synergistic therapy with commonly used antibiotics (such as amoxicillin), providing extra portals for some compounds to enter the bacteria (Krzyzek et al., 2021; Tang et al., 2023). Piperine, an alkaloid present in black pepper, has shown a diminution of *H. pylori* motility as well as adhesion to gastric cells, contributing to the suppression of *H. pylori* growth (Tharmalingam et al., 2014).

Like other natural products, alkaloids can also exert diverse anti-inflammatory effects, including the reduction of certain proinflammatory cytokines (IL-2, IL-6, IL-17, CXCL1) (Tang et al., 2023), the regulation of macrophage activity (Yang et al., 2021), and the modulation of inflammatory signaling pathways (Alam et al., 2019; Haftcheshmeh et al., 2022). In addition to anti-inflammatory properties, some studies have investigated the gastroprotective effects of alkaloids and reported improvements in the ulcer area of rats, with increased prostaglandin E_2_ (PGE_2_) as well as decreased platelet-activating factor (PAF) (Wang et al., 2017). All of these features may relieve the symptoms and health hazards induced by *H. pylori*.

## 5 The Merits of ethnomedicine and further perspectives

Given that only a limited fraction of plant species have been scientifically studied, there still lies great potential in discovering new promising drugs, which are likely to be included in ethnomedicine and are perceived to be an ideal substitute or supplement for current recommended treatment.

Ethnomedicine distinguishes itself from traditional therapies by its accessibility, relatively affordable price and widespread availability. In addition, many consumers perceive herbal medicine as a more natural and thus safer option than synthetic drugs to address diseases, particularly in regions where traditional medicine has been widely practiced for a long time. Although herbal medicine cannot necessarily avoid adverse effects, evidence has proven the safety advantages of natural products over the traditional therapies of combining multiple antibiotics (Deng et al., 2022; Liu et al., 2022). Some studies have investigated the efficacy and safety of the combination of standard therapy and some ethnomedicine medications, which was demonstrated to have a higher eradication rate and fewer side effects than using an antibiotic-based regimen (Bao et al., 2022). In addition to the eradication efficiency, combination medication possesses the superiority of relieving the symptoms of *H. pylori*-associated gastritis, with the concept of overall adjustment and multitargeting curative effects (Li et al., 2021). Moreover, another appealing reason for adding ethnomedicine to treatments is that pharmaceutical synergism may lessen the likelihood of antibiotic resistance, which can also be attributed to the multiple target effects of ethnomedicine (Ngo et al., 2013; Li et al., 2021). Ultimately, the use of ethnomedicine has noteworthy value in the *H. pylori* eradication of specific populations. For example, it is relatively inappropriate to employ conventional antibiotic-based regimens in vulnerable elderly individuals who would be unable to endure the possible adverse effects of high-dose antibiotics and acid suppression. Thus, ethnomedicine, with milder side effects, is regarded as an ideal therapeutic solution.

Apart from the aforementioned advantages from the patients’ perspective, the screening process for new pharmaceuticals also gains from the higher success rate of natural product libraries and the information provided by ethnomedicine. Natural products already possess biological functions since they are mainly primary and secondary metabolites, which leads to a higher possibility of discovering biologically appropriate compounds in natural product libraries than exploring traditional combinatorial chemistry (Sukuru et al., 2009; Buenz et al., 2018). The screening of polyketide metabolites is a good illustration of the efficiency with a hit rate of 0.3% (more than 20 commercial drugs in just over 7000 known structures), while the hit rate for HTS of synthetic compound libraries is less than 0.001% (Weissman and Leadlay, 2005). Additionally, with its empirical knowledge and historical trials, the ethnomedicine system may also facilitate the identification of bioactive compounds by indicating possible screening directions (Buenz et al., 2018).

With the advances of new technologies such as virtual screening, bioinformatics, and artificial intelligence, bioprospecting is currently equipped with innovative powerful tools and undergoing rapid evolution. For instance, in the work of He et al., virtual screening and molecular modeling were utilized to identify ligands that interact with CagA protein to discover potential natural compound candidates (He et al., 2022a). Bioinformatics approaches, including genomics, proteomics, and metabolomics, are also widely implemented in natural product biosynthesis and production, and some researchers bet on artificial intelligence projects for drug innovation inspiration (Ngo et al., 2013; Merwin et al., 2020; Zhang et al., 2020).

While ethnomedicine may provide valuable insights into potential natural remedies or complementary approaches, there are several challenges associated with the development of ethnomedicine as a treatment for *H. pylori*. It has been generally accepted that the premise of TCM treatment is syndrome differentiation, which is the cornerstone of treatment. However, due to the complexity of TCM syndrome types, it is difficult to develop a widely accepted TCM syndrome type differentiation system at present (Li et al., 2021; Elbehiry et al., 2023). Thus, it is necessary to summarize the experience from current clinical practice and integrate experts’ opinions to form consensus about syndrome differentiation for *H. pylori*-associated diseases. In addition, basic animal experimental studies were also limited by the complexity of TCM syndrome types. Currently, most animal experimental studies on disease-syndrome combinations only have a single syndrome type, which is inconsistent with the complex syndrome type of TCM that is more common in clinical practice. Future studies should explore and develop new research models containing complex disease-syndrome combinations to be in line with the current majority clinical practice. Furthermore, the combination of natural products and Western medicine is becoming increasingly common in modern clinical research. This approach can achieve the goal of increasing efficiency and reducing toxicity, highlighting the advantages of combination therapy. Phytochemicals from ethnomedicine formulas and their compositional herb medicines exhibit great potential for the development of novel anti-*H. pylori* drugs. However, the current investigation of the combination of natural products and Western medicine for treating *H. pylori* is still insufficient. Researchers should try to combine some herbal monomers that have clear chemical structures or active ingredients in formulas to develop novel drugs for treating *H. pylori* infection based on the compatibility principle of ethnomedicine prescriptions (Li et al., 2021). Last, the effectiveness and safety of natural products can vary significantly (Périco et al., 2015), and it is important to consider potential interactions with other medications and any possible side effects. There is currently little clinical trial data regarding their safety and efficacy. Therefore, more multicenter, double-blind, randomized, and controlled clinical trials should be conducted to validate the effectiveness and safety of these natural product prescriptions in the treatment of *H. pylori*. Well-designed clinical trials and experimental studies would be beneficial for facilitating a better understanding of the mechanism of natural products and promoting the modernization of natural products in the treatment of *H. pylori*-associated diseases (Yang et al., 2023).

## 6 Summary

Confronting the challenge presented by *H. pylori* antibiotic resistance, ethnomedicine is a particularly noteworthy choice of complementary therapy. The scientists and practitioners should continue working collaboratively to bring this ancient wisdom up-to-date and make full use of it in *H. pylori* eradication. In this review, we comprehensively summarized the anti-*H. pylori* function and mechanisms of natural products, and analyzed the therapeutic advantages of incorporating ethnomedicine into anti-*H. pylori* regimens, including safety, accessibility, efficiency and restriction of antibiotic resistance. Natural products can also provide clues for novel antimicrobial drug discovery. This review can provide insights into the development of natural products and expand the therapeutic options available for *H. pylori* eradication. However, there is still some limitation. The language of the included studies was limited to English, so some potential eligible studies published in other languages might be neglected.

## Author contributions

RD: Data curation, Formal analysis, Investigation, Methodology, Software, Supervision, Validation, Visualization, Writing – original draft, Writing – review & editing. XC: Conceptualization, Data curation, Formal analysis, Investigation, Validation, Visualization, Writing – original draft, Software. SZ: Investigation, Writing – review & editing, Methodology, Validation. QZ: Investigation, Project administration, Supervision, Writing – review & editing, Resources. YS: Conceptualization, Funding acquisition, Resources, Supervision, Writing – review & editing, Methodology, Project administration.
